# *N*-(4-Methoxy­phen­yl)pivalamide

**DOI:** 10.1107/S1600536809029535

**Published:** 2009-08-08

**Authors:** Aamer Saeed, Shahid Hussain, Mahira Batool, Ulrich Flörke

**Affiliations:** a Department of Chemistry, Quaid-i-Azam University, Islamabad 45320, Pakistan; b Department Chemie, Fakultät für Naturwissenschaften, Universität Paderborn, Warburgerstrasse 100, D-33098 Paderborn, Germany

## Abstract

In the title mol­ecule, C_12_H_17_NO_2_, the amide (N—C=O) plane is oriented at an angle of 33.9 (1)° with respect to the aromatic ring. This is accompanied by an intra­molecular C—H⋯O hydrogen bond. The meth­oxy group lies almost in the plane of the benzene ring [C–O–C–C torsion angle = 2.7 (2)°]. In the crystal structure, inter­molecular N—H⋯O hydrogen bonds link the mol­ecules into chains along [010].

## Related literature

For details of the biological activity of benzanilides, see: Olsson *et al.* (2002 ▶); Lindgren *et al.* (2001 ▶); Calderone *et al.* (2006 ▶). For the use of benzamides in organic synthesis, see: Zhichkin *et al.* (2007 ▶); Beccalli *et al.* (2005 ▶). For related structures see: Gowda *et al.* (2007*a* ▶,*b* ▶); Saeed *et al.* (2008 ▶). For a description of the Cambridge Structural Database, see: Allen (2002 ▶).
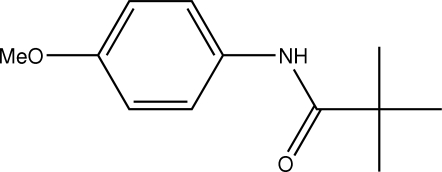


## Experimental

### 

#### Crystal data

C_12_H_17_NO_2_
*M* *_r_* = 207.27
Orthorhombic, 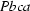

*a* = 9.5547 (13) Å
*b* = 10.0657 (15) Å
*c* = 24.575 (4) Å
*V* = 2363.5 (6) Å^3^
*Z* = 8
Mo *K*α radiation
μ = 0.08 mm^−1^
*T* = 120 K
0.40 × 0.25 × 0.20 mm

#### Data collection

Bruker SMART APEX diffractometer
Absorption correction: multi-scan (*SADABS*; Sheldrick, 2004 ▶) *T*_min_ = 0.969, *T*_max_ = 0.984
17959 measured reflections
2817 independent reflections
2158 reflections with *I* > 2σ(*I*)
*R*_int_ = 0.053

#### Refinement

*R*[*F* ^2^ > 2σ(*F* ^2^)] = 0.048
*wR*(*F* ^2^) = 0.120
*S* = 1.02
2817 reflections
137 parameters
H-atom parameters constrained
Δρ_max_ = 0.32 e Å^−3^
Δρ_min_ = −0.20 e Å^−3^

### 

Data collection: *SMART* (Bruker, 2002 ▶); cell refinement: *SAINT* (Bruker, 2002 ▶); data reduction: *SAINT*; program(s) used to solve structure: *SHELXS97* (Sheldrick, 2008 ▶); program(s) used to refine structure: *SHELXL97* (Sheldrick, 2008 ▶); molecular graphics: *SHELXTL* (Sheldrick, 2008 ▶); software used to prepare material for publication: *SHELXTL*.

## Supplementary Material

Crystal structure: contains datablocks global, I. DOI: 10.1107/S1600536809029535/fl2254sup1.cif

Structure factors: contains datablocks I. DOI: 10.1107/S1600536809029535/fl2254Isup2.hkl

Additional supplementary materials:  crystallographic information; 3D view; checkCIF report

## Figures and Tables

**Table 1:** Hydrogen-bond geometry (Å, °)

*D*—H⋯*A*	*D*—H	H⋯*A*	*D*⋯*A*	*D*—H⋯*A*
N1—H1*A*⋯O1^i^	0.88	2.09	2.9382 (15)	160
C3—H3*A*⋯O1	0.95	2.45	2.9063 (17)	109

Symmetry code: (i) 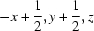.

**Fig. 1.**
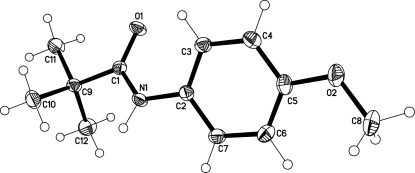
Molecular structure of title compound. Displacement ellipsoids are drawn at the 50% probability level.

**Fig. 2.**
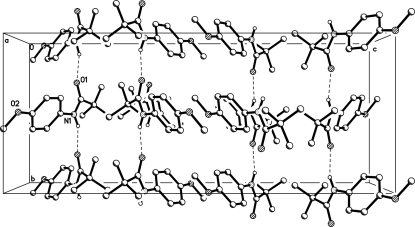
Crystal packing viewed along [100] with intermolecular hydrogen bonding pattern indicated as dashed lines. H-atoms not involved in hydrogen bonding are omitted.

## supplementary crystallographic information

### Comment

N-substituted benzamides are well known anticancer compounds and the mechanism of action for N-substituted benzamide-induced apoptosis has been studied, using declopramide as a lead compound (Olsson *et al.*, 2002). N-substituted benzamides inhibit the activity of nuclear factor- B and nuclear factor of activated T cells activity while inducing activator protein 1 activity in T lymphocytes (Lindgren *et al.*, 2001). Heterocyclic analogs of benzanilide derivatives are potassium channel activators (Calderone *et al.*, 2006). *N*-Alkylated 2-nitrobenzamides are intermediates in the synthesis of dibenzo[b,e][1,4]diazepines (Zhichkin *et al.*, 2007) and *N*-Acyl-2-nitrobenzamides are precursors of 2,3-disubstitued 3*H*-quinazoline-4-ones (Beccalli *et al.*, 2005).

The molecular structure of the title compound (Fig. 1)is closely related to two other compounds (Gowda *et al.*, 2007*a*; 2007*b*) that exhiibt a methyl or chloro ligand instead of the methoxy group (CCDC refcodes HIDVOG and QIFKAS (Allen, 2002)), respectively. The dihedral angles between the benzene ring and the amide group are 33.9 (1)° for the title molecule and 32.8 (1)° for HIDVOG and 31.3 (1)° for QIFKAS, respectively. The C8–O2–C5–C6 torsion angle of 2.7 (2)° shows almost in-plane orientation of the methoxy group with respect to the aromatic ring. In the cystal structure, intermolecular N–H···O hydrogen bonds (Table 1) link the molecules into infinite chains along the direction (Fig. 2). A somewhat longer intramolecular C–H···O hydrogen bond is associated with the twist of the amide plane.

### Experimental

Pivaloyl chloride (1 mmol) in CHCl_3_ was treated with 4-methoxyaniline (3.5 mmol) under a nitrogen atmosphere at reflux for 5 h. Upon cooling, the reaction mixture was diluted with CHCl_3_ and washed consecutively with 1 *M* aq HCl and saturated aq NaHCO_3_. The organic layer was dried over anhydrous sodium sulfate and concentrated under reduced pressure. Crystallization of the residue in methanol afforded the title compound (84%) as white needles: Anal. calcd. for C_12_H_17_NO_2_: C 59.54, H 8.27, N 6.76%; found: 59.51, H 8.31, N 6.82%.

### Refinement

Hydrogen atoms were located in difference syntheses, refined at idealized positions riding on the C (C–H = 0.95–0.99 Å) or N (N–H = 0.88 Å) atoms with isotropic displacement parameters *U*_iso_(H) = 1.2U(C_eq_ / N_eq_) and 1.5U(C_methyl_). All methyl hydrogen atoms were allowed to rotate but not to tip.

### Crystal data

**Table:**

C_12_H_17_NO_2_	*F*(000) = 896
*M**_r_* = 207.27	*D*_x_ = 1.165 Mg m^−^^3^
Orthorhombic, *P**b**c**a*	Mo *K*α radiation, λ = 0.71073 Å
Hall symbol: -P 2ac 2ab	Cell parameters from 778 reflections
*a* = 9.5547 (13) Å	θ = 2.7–27.0°
*b* = 10.0657 (15) Å	µ = 0.08 mm^−^^1^
*c* = 24.575 (4) Å	*T* = 120 K
*V* = 2363.5 (6) Å^3^	Block, colourless
*Z* = 8	0.40 × 0.25 × 0.20 mm

### Data collection

**Table:**

Bruker SMART APEX diffractometer	2817 independent reflections
Radiation source: sealed tube	2158 reflections with *I* > 2σ(*I*)
graphite	*R*_int_ = 0.053
φ and ω scans	θ_max_ = 27.9°, θ_min_ = 1.7°
Absorption correction: multi-scan (*SADABS*; Sheldrick, 2004)	*h* = −9→12
*T*_min_ = 0.969, *T*_max_ = 0.984	*k* = −13→13
17959 measured reflections	*l* = −31→32

### Refinement

**Table:**

Refinement on *F*^2^	Primary atom site location: structure-invariant direct methods
Least-squares matrix: full	Secondary atom site location: difference Fourier map
*R*[*F*^2^ > 2σ(*F*^2^)] = 0.048	Hydrogen site location: difference Fourier map
*wR*(*F*^2^) = 0.120	H-atom parameters constrained
*S* = 1.02	*w* = 1/[σ^2^(*F*_o_^2^) + (0.0527*P*)^2^ + 0.8944*P*] where *P* = (*F*_o_^2^ + 2*F*_c_^2^)/3
2817 reflections	(Δ/σ)_max_ < 0.001
137 parameters	Δρ_max_ = 0.32 e Å^−^^3^
0 restraints	Δρ_min_ = −0.19 e Å^−^^3^

### Special details

**Table:**

Geometry. All e.s.d.'s (except the e.s.d. in the dihedral angle between two l.s. planes) are estimated using the full covariance matrix. The cell e.s.d.'s are taken into account individually in the estimation of e.s.d.'s in distances, angles and torsion angles; correlations between e.s.d.'s in cell parameters are only used when they are defined by crystal symmetry. An approximate (isotropic) treatment of cell e.s.d.'s is used for estimating e.s.d.'s involving l.s. planes.
Refinement. Refinement of *F*^2^ against ALL reflections. The weighted *R*-factor *wR* and goodness of fit *S* are based on *F*^2^, conventional *R*-factors *R* are based on *F*, with *F* set to zero for negative *F*^2^. The threshold expression of *F*^2^ > σ(*F*^2^) is used only for calculating *R*-factors(gt) *etc*. and is not relevant to the choice of reflections for refinement. *R*-factors based on *F*^2^ are statistically about twice as large as those based on *F*, and *R*- factors based on ALL data will be even larger.

### Fractional atomic coordinates and isotropic or equivalent isotropic displacement parameters (Å^2^)

**Table:**

	*x*	*y*	*z*	*U*_iso_*/*U*_eq_	
O1	0.23114 (10)	0.78822 (9)	0.15214 (4)	0.0261 (2)	
O2	−0.30713 (11)	0.99245 (12)	0.02439 (5)	0.0353 (3)	
N1	0.18414 (12)	1.00795 (11)	0.14651 (5)	0.0221 (3)	
H1A	0.2122	1.0875	0.1566	0.027*	
C1	0.26370 (14)	0.90357 (13)	0.16238 (5)	0.0196 (3)	
C2	0.05959 (14)	1.00104 (13)	0.11504 (5)	0.0203 (3)	
C3	−0.03786 (15)	0.89929 (13)	0.12087 (6)	0.0233 (3)	
H3A	−0.0213	0.8293	0.1460	0.028*	
C4	−0.15869 (15)	0.90042 (14)	0.09003 (6)	0.0264 (3)	
H4A	−0.2249	0.8307	0.0940	0.032*	
C5	−0.18439 (15)	1.00250 (15)	0.05324 (6)	0.0253 (3)	
C6	−0.08911 (16)	1.10480 (14)	0.04785 (6)	0.0270 (3)	
H6A	−0.1068	1.1757	0.0233	0.032*	
C7	0.03299 (15)	1.10322 (13)	0.07865 (6)	0.0249 (3)	
H7A	0.0991	1.1731	0.0747	0.030*	
C8	−0.33901 (18)	1.09944 (18)	−0.01185 (7)	0.0375 (4)	
H8A	−0.3448	1.1826	0.0088	0.056*	
H8B	−0.4289	1.0823	−0.0298	0.056*	
H8C	−0.2652	1.1067	−0.0394	0.056*	
C9	0.39771 (15)	0.93998 (13)	0.19354 (6)	0.0227 (3)	
C10	0.36095 (17)	1.02511 (15)	0.24320 (7)	0.0313 (4)	
H10A	0.4468	1.0479	0.2629	0.047*	
H10B	0.2983	0.9752	0.2672	0.047*	
H10C	0.3143	1.1067	0.2312	0.047*	
C11	0.46851 (17)	0.81265 (15)	0.21301 (7)	0.0337 (4)	
H11A	0.5542	0.8351	0.2329	0.050*	
H11B	0.4920	0.7571	0.1816	0.050*	
H11C	0.4048	0.7640	0.2371	0.050*	
C12	0.49651 (16)	1.01588 (16)	0.15543 (7)	0.0328 (4)	
H12A	0.5824	1.0391	0.1751	0.049*	
H12B	0.4504	1.0972	0.1428	0.049*	
H12C	0.5199	0.9599	0.1241	0.049*	

### Atomic displacement parameters (Å^2^)

**Table:**

	*U*^11^	*U*^22^	*U*^33^	*U*^12^	*U*^13^	*U*^23^
O1	0.0257 (5)	0.0146 (5)	0.0380 (6)	−0.0001 (4)	−0.0074 (4)	−0.0020 (4)
O2	0.0262 (6)	0.0441 (7)	0.0356 (6)	−0.0021 (5)	−0.0122 (5)	0.0052 (5)
N1	0.0226 (6)	0.0141 (5)	0.0297 (6)	−0.0008 (4)	−0.0065 (5)	−0.0017 (5)
C1	0.0211 (7)	0.0177 (6)	0.0201 (6)	0.0008 (5)	−0.0003 (5)	0.0001 (5)
C2	0.0201 (7)	0.0184 (6)	0.0223 (6)	0.0026 (5)	−0.0026 (5)	−0.0024 (5)
C3	0.0237 (7)	0.0197 (6)	0.0266 (7)	0.0008 (5)	−0.0012 (6)	0.0018 (5)
C4	0.0226 (7)	0.0253 (7)	0.0315 (8)	−0.0035 (6)	−0.0006 (6)	−0.0012 (6)
C5	0.0195 (7)	0.0324 (8)	0.0240 (7)	0.0026 (6)	−0.0019 (5)	−0.0040 (6)
C6	0.0275 (8)	0.0269 (7)	0.0267 (8)	0.0040 (6)	−0.0023 (6)	0.0056 (6)
C7	0.0241 (7)	0.0201 (7)	0.0307 (8)	−0.0009 (5)	−0.0029 (6)	0.0026 (6)
C8	0.0318 (9)	0.0506 (10)	0.0301 (9)	0.0057 (8)	−0.0106 (7)	0.0047 (7)
C9	0.0230 (7)	0.0172 (6)	0.0278 (7)	0.0001 (5)	−0.0058 (6)	0.0009 (5)
C10	0.0346 (9)	0.0283 (7)	0.0310 (8)	−0.0007 (6)	−0.0095 (7)	−0.0052 (6)
C11	0.0326 (9)	0.0231 (7)	0.0453 (10)	0.0035 (6)	−0.0177 (7)	0.0017 (7)
C12	0.0209 (7)	0.0365 (8)	0.0409 (9)	−0.0025 (6)	−0.0036 (7)	0.0071 (7)

### Geometric parameters (Å, °)

**Table:**

O1—C1	1.2282 (16)	C7—H7A	0.9500
O2—C5	1.3741 (17)	C8—H8A	0.9800
O2—C8	1.4304 (19)	C8—H8B	0.9800
N1—C1	1.3542 (17)	C8—H8C	0.9800
N1—C2	1.4209 (17)	C9—C11	1.5262 (19)
N1—H1A	0.8800	C9—C10	1.532 (2)
C1—C9	1.5362 (19)	C9—C12	1.534 (2)
C2—C7	1.3866 (19)	C10—H10A	0.9800
C2—C3	1.3916 (19)	C10—H10B	0.9800
C3—C4	1.381 (2)	C10—H10C	0.9800
C3—H3A	0.9500	C11—H11A	0.9800
C4—C5	1.391 (2)	C11—H11B	0.9800
C4—H4A	0.9500	C11—H11C	0.9800
C5—C6	1.381 (2)	C12—H12A	0.9800
C6—C7	1.391 (2)	C12—H12B	0.9800
C6—H6A	0.9500	C12—H12C	0.9800
			
C5—O2—C8	116.59 (12)	O2—C8—H8C	109.5
C1—N1—C2	126.08 (11)	H8A—C8—H8C	109.5
C1—N1—H1A	117.0	H8B—C8—H8C	109.5
C2—N1—H1A	117.0	C11—C9—C10	108.76 (12)
O1—C1—N1	122.16 (13)	C11—C9—C12	109.69 (13)
O1—C1—C9	122.60 (12)	C10—C9—C12	110.42 (12)
N1—C1—C9	115.24 (11)	C11—C9—C1	108.99 (11)
C7—C2—C3	119.32 (13)	C10—C9—C1	109.84 (12)
C7—C2—N1	117.92 (12)	C12—C9—C1	109.12 (11)
C3—C2—N1	122.71 (12)	C9—C10—H10A	109.5
C4—C3—C2	119.79 (13)	C9—C10—H10B	109.5
C4—C3—H3A	120.1	H10A—C10—H10B	109.5
C2—C3—H3A	120.1	C9—C10—H10C	109.5
C3—C4—C5	120.70 (13)	H10A—C10—H10C	109.5
C3—C4—H4A	119.6	H10B—C10—H10C	109.5
C5—C4—H4A	119.6	C9—C11—H11A	109.5
O2—C5—C6	124.62 (13)	C9—C11—H11B	109.5
O2—C5—C4	115.58 (13)	H11A—C11—H11B	109.5
C6—C5—C4	119.80 (13)	C9—C11—H11C	109.5
C5—C6—C7	119.50 (13)	H11A—C11—H11C	109.5
C5—C6—H6A	120.3	H11B—C11—H11C	109.5
C7—C6—H6A	120.3	C9—C12—H12A	109.5
C2—C7—C6	120.88 (13)	C9—C12—H12B	109.5
C2—C7—H7A	119.6	H12A—C12—H12B	109.5
C6—C7—H7A	119.6	C9—C12—H12C	109.5
O2—C8—H8A	109.5	H12A—C12—H12C	109.5
O2—C8—H8B	109.5	H12B—C12—H12C	109.5
H8A—C8—H8B	109.5		
			
C2—N1—C1—O1	−2.7 (2)	O2—C5—C6—C7	−178.91 (13)
C2—N1—C1—C9	176.75 (12)	C4—C5—C6—C7	1.2 (2)
C1—N1—C2—C7	−145.49 (14)	C3—C2—C7—C6	−0.3 (2)
C1—N1—C2—C3	37.0 (2)	N1—C2—C7—C6	−177.94 (13)
C7—C2—C3—C4	0.7 (2)	C5—C6—C7—C2	−0.6 (2)
N1—C2—C3—C4	178.23 (13)	O1—C1—C9—C11	−5.65 (19)
C2—C3—C4—C5	−0.2 (2)	N1—C1—C9—C11	174.89 (13)
C8—O2—C5—C6	−2.7 (2)	O1—C1—C9—C10	−124.71 (14)
C8—O2—C5—C4	177.17 (13)	N1—C1—C9—C10	55.83 (16)
C3—C4—C5—O2	179.31 (13)	O1—C1—C9—C12	114.12 (15)
C3—C4—C5—C6	−0.8 (2)	N1—C1—C9—C12	−65.34 (15)

### Hydrogen-bond geometry (Å, °)

**Table:**

*D*—H···*A*	*D*—H	H···*A*	*D*···*A*	*D*—H···*A*
N1—H1A···O1^i^	0.88	2.09	2.9382 (15)	160
C3—H3A···O1	0.95	2.45	2.9063 (17)	109

Symmetry codes: (i) −*x*+1/2, *y*+1/2, *z*.
